# Characteristics of Keratoconus Patients at a Tertiary Eye Center in India

**Published:** 2011-04

**Authors:** Vinay B Agrawal

**Affiliations:** Clear Vision Eye Center, Mumbai, India

**Keywords:** Keratoconus, Characteristics, Demography, India

## Abstract

**Purpose:**

To evaluate the presentation and characteristics of patients with keratoconus at a tertiary eye care center in Mumbai, India.

**Methods:**

This single center, non-comparative, retrospective cohort analysis was performed on patients with keratoconus who presented to the Clear Vision Eye Center clinic from April 2007 to March 2009. Data was collected to characterize correlations among visual acuity, corneal biomicroscopic findings, and refractive and topographic findings in keratoconus.

**Results:**

Records of 274 patients including 189 male and 85 female subjects with mean age of 20.1±3.5 (range, 13 to 29) years at the time of diagnosis were assessed. There was history of skin allergy in 73 (26.6%), symptomatic ocular allergy in 67 (24.45%) and asthma in 31 (11.31%) patients. The most frequent corneal sign was Fleischer’s ring which was observed in 81% of cases. Corneal topography revealed mean simK (simulated keratometry) of 53.3±6.1 (range, 41.2 to 69.0) diopters. Corneal topography analysis with the Cone Location Magnitude Index disclosed the presence of inferior cones in 93% of patients.

**Conclusion:**

This group of patients had younger age at presentation and more severe keratoconus as compared to western populations; contact lenses were used only in a minority of patients.

## INTRODUCTION

Keratoconus is a relatively common ectatic corneal condition causing significant visual disability. It is characterized by progressive irregular myopic astigmatism with central corneal thinning and protrusion.^1^ Keratoconus affects both eyes in the majority of cases but may be markedly asymmetric. The Collaborative Longitudinal Evaluation of Keratoconus (CLEK) Group has presented findings related to keratoconus in a large cohort of patients from diverse ethnicities in the USA.^2^ Similarly the Dundee University Group has published a longitudinal study on a more homogenous population from Scotland.^3^ However, characteristics of keratoconus in Asian populations seem to vary from those reported from other ethnic groups. In a population based cohort analysis, the Central India Eye and Medical Study reported the prevalence of keratoconus in central India to be 1.4%.^4^ The incidence of keratoconus has been shown to range from 1 in 4,000 to 5,100 persons per year in Asian populations.^5^,^6^

The purpose of this study was to characterize correlations among visual acuity, and biomicroscopic, refractive and topographic findings in keratoconus. The study consisted of an Asian-Indian group of patients attending a single tertiary care center in India.

## METHODS

This retrospective observational study was based at the cornea service of a tertiary eye care center with a special focus on keratoconus in Mumbai, India. A total of 293 patients diagnosed with keratoconus were identified and evaluated during a 2 year period from April 2007 to March 2009. Complete records were available for 274 patients. All subjects demonstrated corneal signs of keratoconus. Corneal topography (Keratron Scout, Optikon, Rome, Italy) supported the diagnosis of keratoconus in all subjects. Cone Location and Magnitude Index (CLMI)^7^ was used to detect the presence or absence of keratoconus patterns. Corneal thickness was calculated at the cone apex as suggested by CLMI on topography using ultrasonic pachymetry (Micro Medical Technologies, Calabasas, CA, USA). The diagnosis was made by a consultant trained in cornea subspecialty.

## RESULTS

**Figure 1:**
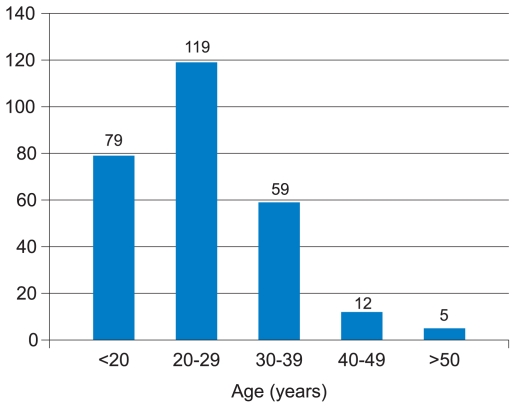
Age distribution of the study population.

The study included 189 (68.9%) male and 85 (31.1%) female subjects. Age distribution is detailed in figure 1. A sizeable proportion of cases (43.4%) were 20 to 29 years of age followed by subjects less than 20 years (28.8%). Mean age was 21.4 ± 10.1 (range, 13 to 54) years at the time of the study and 20.1 ± 3.5 (range, 13 to 29) years at the time of diagnosis.

Educational levels of the patients are shown in Table 1. A significant number of patients (58.7%) had completed at least graduation.

No patient had history of cardiac or joint problems. There were skin allergies in 73 (26.6%), symptomatic ocular allergies (vernal catarrh) in 67 (24.5%), and asthma in 31 (11.3%) patients. Only 3 (1.1%) patients had positive family history of keratoconus. Nine (3.3%) patients had undergone penetrating keratoplasty in one eye at the time of assessment.

The type of vision correction used by our patients is shown in Table 2. Most subjects (46.3%) used glasses as the only means for vision correction. Contact lenses were solely used by 60 (21.9%) patients and 48 individuals (17.5%) used glasses some of the time in addition to contact lenses. Thirty-nine (14.2%) patients used no form of optical correction.

Snellen visual acuity in subjects using spectacles (best manifest refraction) and among contact lens wearers is shown in table 3. Visual acuity was 6/12 or better in 59% of contact lens wears and 30% of spectacle wearers.

The most frequently observed corneal sign was Fleischer’s ring (81%). Only 7% of patients had cones but no other corneal signs. Vogt’s striae and corneal scarring were found to be more prevalent with increasing severity of the disease (Table 4).

Bilateral keratoconus was detected in all subjects. Corneal topography showed mean simK (simulated keratometry) of 53.3 ± 6.1 (range, 41.2 to 69) diopters (D). CLMI showed the presence of an inferior cone in 93% of eyes; 1% of eyes were indeterminate in terms of classification of cone type. Mean corneal thickness was 409 (range, 296 to 534) μm; corneal thickness less than 480 μm was present in 61% of studied eyes.

## DISCUSSION

Our study reports slitlamp biomicroscopic and demographic characteristics in a cohort of patients with keratoconus at a tertiary eye care center in India. There was a higher percentage of male subjects indicating a trend toward earlier diagnosis in this gender. Mean age at the time of diagnosis of keratoconus is typically in the second decade of life.^8^ In our series, mean age at diagnosis was 20 years which is similar to other Asian populations^5^,^6^ but much lower than that of Caucasian populations.^2^,^3^ Younger age at diagnosis could mean that the condition has earlier onset and faster progression in Asian populations reflecting variability of the disease process.

Similar to the DUSKS study,^3^ there were very few patients over 40 years of age in our series. Keratoconus patients demonstrate a period of stability 8 to 12 years after diagnosis,^9^ thus, most of them may seek care with local ophthalmologists/contact lens practitioners. These subjects are thus likely to be underrepresented in a sample from a tertiary care center which would expectedly handle patients seeking further management.

Disease severity was found to be positively associated with an increasing rate of slitlamp biomicroscopic signs (Vogt’s striae, Fleischer’s ring and corneal scarring). Our findings support the clinical impression that higher keratometric readings are associated with a higher prevalence of one or more slitlamp signs of keratoconus. However, a diagnosis can also be made in the absence of corneal signs. This is reflected by the fact that 7% of patients with a diagnosis of keratoconus had no corneal signs on slitlamp evaluation. Our findings are different from the CLEK^2^ and DUSKS^3^ studies which reported corresponding figures of 14% and 15%, respectively. One may thus imply that we are seeing more severe cases of keratoconus in India as compared to Europe and America.

The type of vision correction used by patients in this study reflects the lack of awareness about keratoconus and its treatment modalities, although one should reserve the possibility of lack of access to appropriate care. About 15% of our patients were not using any visual correction and almost half of them were using glasses. Only 40% of our patients were using contact lenses as compared to 75% of patients in the CLEK study.^10^ It is noteworthy that the DUSK^3^ study reported only 9% of patients to be wearing contact lenses (three fourths were using soft lenses) prior to being seen at the university. These differences may be attributed to the varied medical care systems in the countries where these studies were performed. In India, where the most prominent form of treatment available to the population is fee-for-care based, patients usually come to a tertiary care center only if they are unable to get adequate care at the primary or secondary level. This should skew the incidence to the more severe population, yet only less than half of the patients were using contact lenses. Considering the fact that India is a developing country with limited resources, we believe this may reflect the lack of ability among practitioners to fit these challenging patients with rigid gas permeable contact lenses or the lack of availability of trained personnel for these complex fittings.

Visual acuity using spectacles (best manifest refraction) was 6/12 or better in 30% of patients in this study which is much less than the 58% rate reported in the CLEK study^11^ and may reflect more advanced disease in our series. Alternatively it may be interpreted that our patients tend to present with more advanced disease. It is a general observation that the use of contact lenses is very useful in restoration of vision in keratoconus patients. The importance of contact lenses is further emphasized by the fact that although only 40% of our patients were contact lens wearers, they comprised 59% of subjects with visual acuity of 6/12 or better which is almost twice the rate of individuals not using contact lenses.

Atopy has been proposed as an important factor in the etiology of keratoconus and an association has been shown.^12^ In our study only a quarter of patients had systemic or ocular allergies. This is much less than the rate reported in studies from the west and such a discrepancy has been noted in studies comparing keratoconus patients of Asian or Caucasian origin.^5^ Similarly, the association with various cardiac and joint disorders was absent in our patients. This could reflect lack of detection, low level of penetration of associated diseases, an evolving disease process or different underlying mechanisms of keratoconus in the Asian-Indian population.

In our study only 3 patients reported a family history of keratoconus. These relatives were not examined in our study and thus information was not complete. Lack of awareness about the ocular condition of first or second degree relatives is also a possibility; some cases with positive family history may have been missed which is an inherent problem due to the retrospective nature of the study.

It is possible that keratoconus represents a spectrum of conditions rather than a single identifiable disease. The condition may be the end result of several different pathological processes. It is believed that in keratoconus, an unidentified primary event which may be under genetic control,^13^ triggers a breakdown of the “glue” that stabilizes collagen fibrils, thus predisposing to lamellar or fibrillar slippage.^14^ If this is correct, it may be worthwhile to study specific ethnic groups with keratoconus or those with a specified type of keratoconus.

There are certain limitations to our study. First, it is retrospective in nature and relies on clinical case records. Second, this study looked only at patients who presented to our eye care facility, usually with a preexisting diagnosis of keratoconus. Thus more severe forms of the disease may have been over-represented due to selection bias. Further family based studies are required to identify possible underlying genetic factors.

## Figures and Tables

**Table 1:** Educational level of the patients

Education	No.	%
Primary School	2	0.7
Secondary School (10 years of school)	84	30.6
Senior Secondary School (equivalent to high school)	27	9.8
Graduation (equal to 15 years of education)	130	47.4
Post-graduation	31	11.3

**Table 2:** Type of vision correction used by the patients

Type of Correction	No.	%
Unaided	39	14.23
Glasses	127	46.35
Contact lenses	60	21.89
Glasses and contact lenses	48	17.51

Total	274	100

**Table 3:** Distribution of visual acuity with best manifest refraction and contact lenses

Visual acuity	with best manifest refraction	with contact lenses
6/6 or better	6%	19%
6/9 to 6/12	24%	40%
6/18 to 6/24	56%	34%
≤ 6/36	14%	7%

Total	100%	100%

**Table 4:** Correlation between keratometric readings and slitlamp findings in eyes with keratoconus

Disease severity*	Fleischer’s ring (%)	Vogt’s striae (%)	Corneal scarring (%)
Mild (<45D)	18/66 (27.27)	12/66 (18.18)	2/66 (3.03)
Moderate (45–52D)	239/396 (60.35)	186/396 (46.96)	48/396 (12.12)
Advanced (>52D)	63/86 (73.25)	60/86 (69.76)	26/86 (30.23)

* based on simK in diopters (D)
